# Leptomeningeal metastasis from large-cell neuroendocrine carcinoma of the cervix: a case report and literature review

**DOI:** 10.3389/fonc.2026.1734305

**Published:** 2026-02-13

**Authors:** Peng Xu, Zhen-Jiang Wang, Xin-Mei Dang, Bao-Yu Zhu, Zhang-Cai Zheng

**Affiliations:** Gansu Provincial Maternal and Child-care Hospital (Gansu Provincial Central Hospital), Lanzhou, China

**Keywords:** cervical cancer, immune checkpoint inhibitors, leptomeningeal metastasis, neuroendocrine carcinoma of the cervix, pemetrexed

## Abstract

Leptomeningeal metastasis (LM) from neuroendocrine carcinoma of the cervix (NECC) is extremely rare, with only five cases reported in the literature. Immune checkpoint inhibitors (ICIs) have the standard first-line treatment for metastatic cervical carcinoma and have been reported to improve intracranial response and survival in several types of cancer. A 50-year-old woman initially received pelvic radiation and chemotherapy for cervical cancer. At 1 year later, she complained of left breast, supraclavicular, and retroperitoneal lymph node metastases. She received targeted and adjuvant chemotherapy. At 15 months after diagnosis, she received radiotherapy due to nasopharynx and supraclavicular lymph node metastasis as well as treatment with a bispecific antibody targeting PD-1 and CTLA-4, cadonilimab, combined with chemotherapy. At 20 months after diagnosis, she experienced a transient unconsciousness and severe headache, nausea, and vomiting. The cytological demonstration of malignant cells in the cerebrospinal fluid (CSF) confirmed the presence of leptomeningeal metastasis. The therapeutic regimen then consisted of intrathecal chemotherapy combined with oral temozolomide. The authors highlight the diagnosis and treatment of LM from NECC, providing a rare clinical scenario.

## Introduction

Neuroendocrine carcinoma of the cervix (NECC), a rare and aggressive cervical cancer, accounts for a small fraction of cervical cancer cases, with an incidence rate of 0.9%–1.5%. Moreover, over 80% of these cases are small cell neuroendocrine carcinoma of the cervix (SCNECC) (1). Simultaneously, leptomeningeal metastasis (LM) from NECC is even rarer, and the prognosis is poor (2). LM, also known as carcinomatous meningitis, is characterized by the spread or multifocal dissemination of metastatic cancer cells on the leptomeninges, with or without parenchymal brain metastasis. The most common primary cancers that lead to meningeal metastases include breast cancer, lung cancer, melanoma, lymphoma, and leukemia (3, 4). The incidence of LM has been increasing recently, and the diversification of treatment approaches, along with the updating of diagnostic procedures, prolong survival (5, 6). As far as we know, LM from NECC is rarer; only five cases have been reported in the literature (7–11). We added a case of LM from a large cell neuroendocrine carcinoma of the cervix (LCNECC). Our patient had LM without brain parenchyma metastasis. Here we report a 50-year-old woman with metastatic LCNECC who subsequently developed LM after immune checkpoint inhibitor (ICI) treatment combined with chemoradiotherapy. We also review the diagnosis and treatment of LM, describing our regimen as an exploratory and purely palliative approach after ICIs cannot be used due to adverse drug reaction. This case report was prepared in accordance with the CARE (case report) guidelines.

## Case report

**Figure 1:**
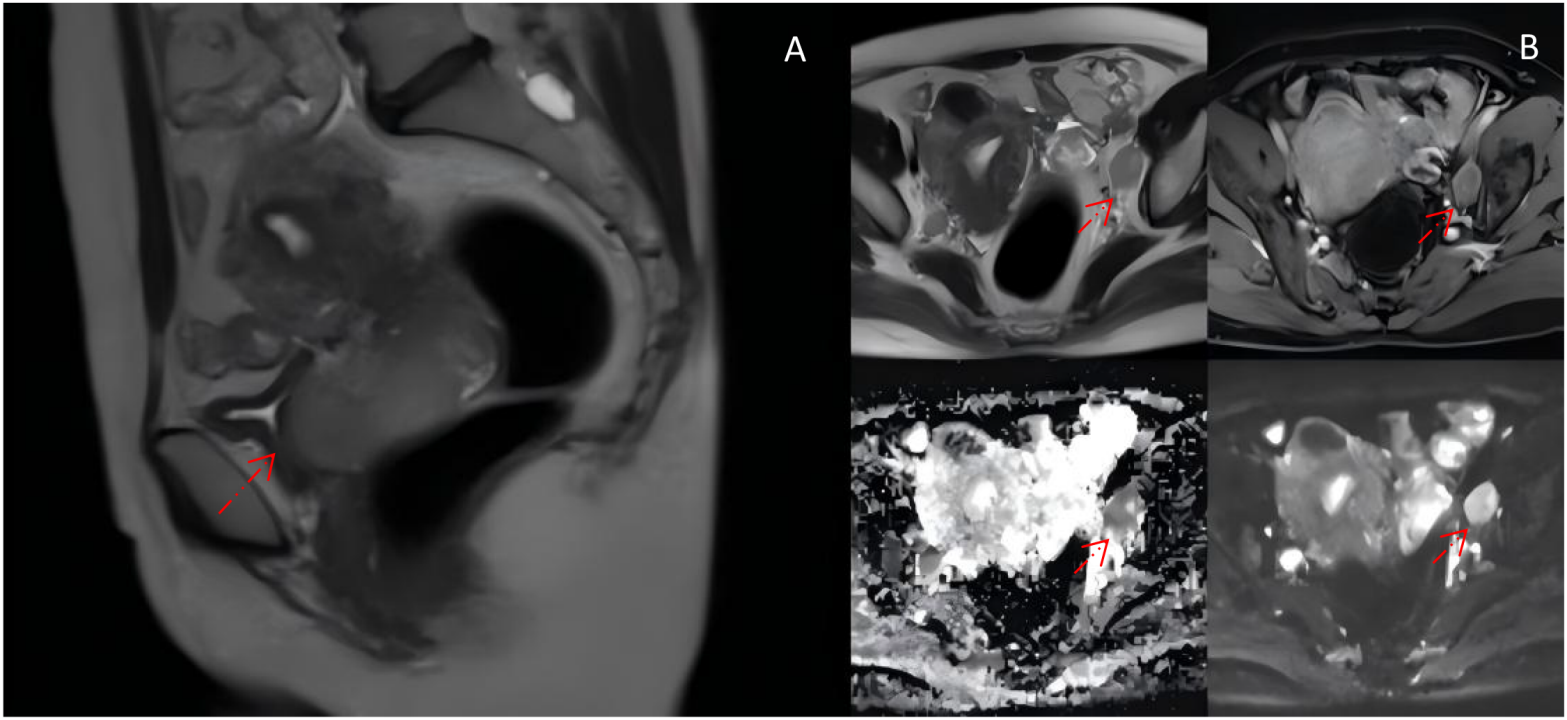
**(A)** T2 magnetic resonance imaging with contrast of the pelvis showing a lesion in the cervix 5.4 × 6.1 (AP diameter) × 6.6 (craniocaudal). **(B)** Initial axial diagnostic MRI showing a metastatic lymph node (arrow) 3.0 × 1.8 (AP diameter) × 3.1 (craniocaudal).

**Figure 2:**
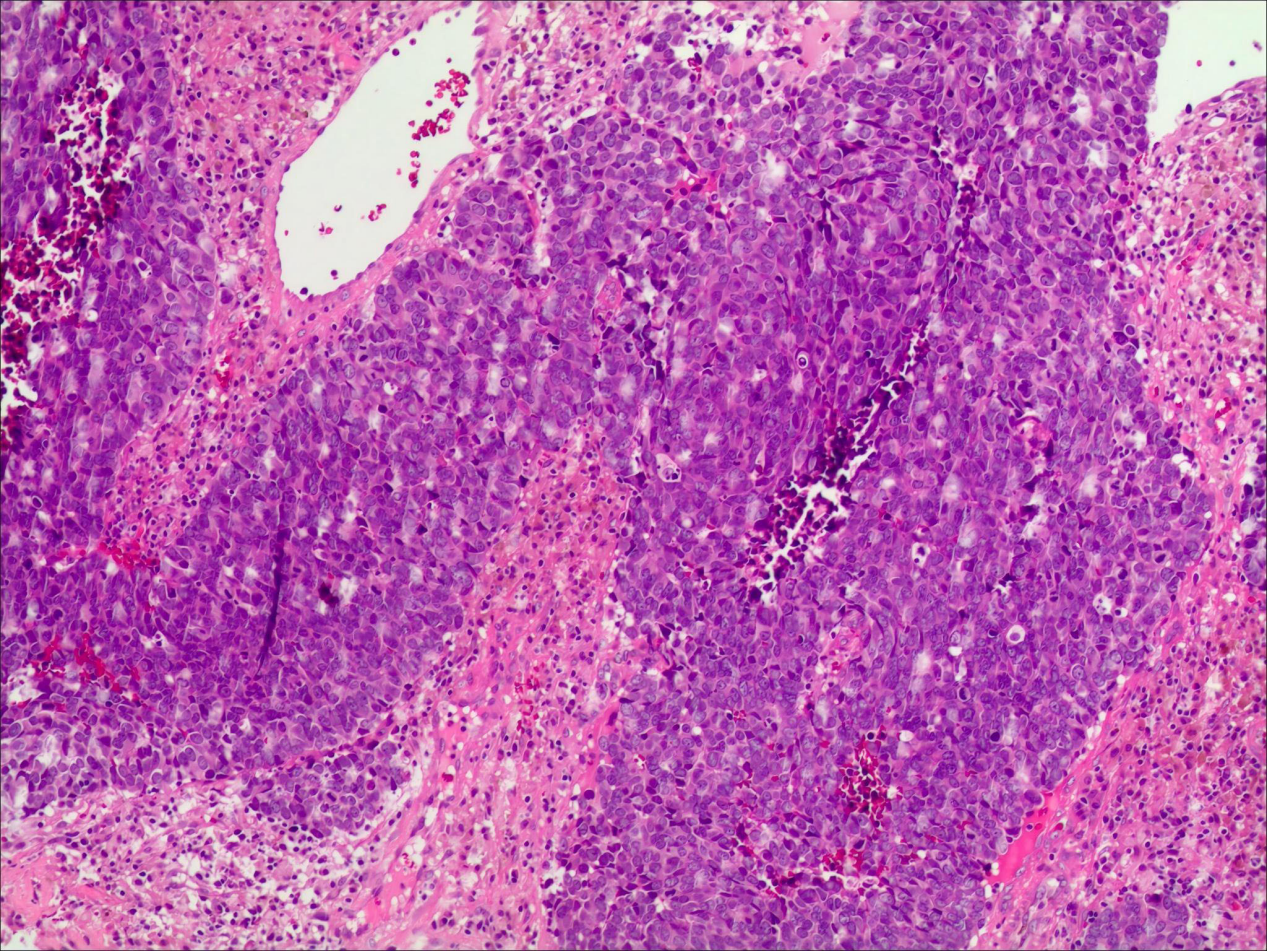
Histopathology slides with immunochemistry for the uterine cervix biopsies (hematoxylin and eosin (H&E), ×100). Microscopically, the tumor cells are large, featuring abundant cytoplasm and vesicular nuclei. Geographic necrosis is identified within the tumor parenchyma.

A 50-year-old woman was initially diagnosed with FIGO 2018 IIIC1r stage LCNECC and HPV16 +. A mass lesion was detected on magnetic resonance imaging (MRI) in the cervix, measuring approximately 5.4 × 6.1 (AP diameter) × 6.6 (craniocaudal). Metastatic lymph nodes are observable in the vicinity of the left iliac artery, measuring approximately 3.0 × 1.8 (AP diameter) × 3.1 (craniocaudal) (**Figure 1**). Biopsy revealed an invasive high-grade neuroendocrine carcinoma, most consistent with large-cell type (**Figure 2**). Immunohistochemical (IHC) staining was positive for P16, Syn, and CD56; negative ßor CK5/6 and CgA; and showed a Ki-67 ndex of 90%. PD-L1 CPS = 5 (paraffin-embedded tumor tissue sections from a core needle biopsy sample: PD-L1 IHC) staining was performed on a 4-um-thick full-face tissue section using the anti-PD-L1 antibody clone 22C3 (Dako, USA; dilution 1:50) on a DAKO Autostainer Link 48 platform. The result was reported as the PD-L1 combined positive score (CPS), defined as (number of PD-L1 membrane-stained tumor cells + number of PD-L1 membrane-stained tumor-associated immune cells)/total number of viable tumor cells × 100. The patient was treated with concurrent cisplatin/etoposide and radiation (XRT) as well as additional brachytherapy.

At 1 year after diagnosis, positron emission tomography (PET) scanning showed that metastases affected the left breast, bilateral supraclavicular, and retroperitoneal lymph nodes. Due to family financial constraints, the patient rejected ICIs but was treated with chemotherapy in the form of four cycles of concurrent cisplatin/etoposide and bevacizumab as well as retroperitoneal metastatic lymph node consolidation radiotherapy with a dose of 3,500 cGy in five fractions.

At 15 months after diagnosis, the patient complained of pharyngeal pain, which worsened after consuming hard or hot food. Pathology immunohistochemistry consultation of the second recurrence was nasopharynx. She received definitive intensity-modulated radiotherapy (IMRT) to local and regional lesions (total dose of 66 Gy/33F, 7 weeks) along with chemotherapy in the form of three cycles of concurrent cisplatin/paclitaxel and ICIs cadonilimab (anti-PD-1 and CTLA-4 antibody, 625 mg on day 1) administered intravenously every 3-week cycle.

**Figure 3:**
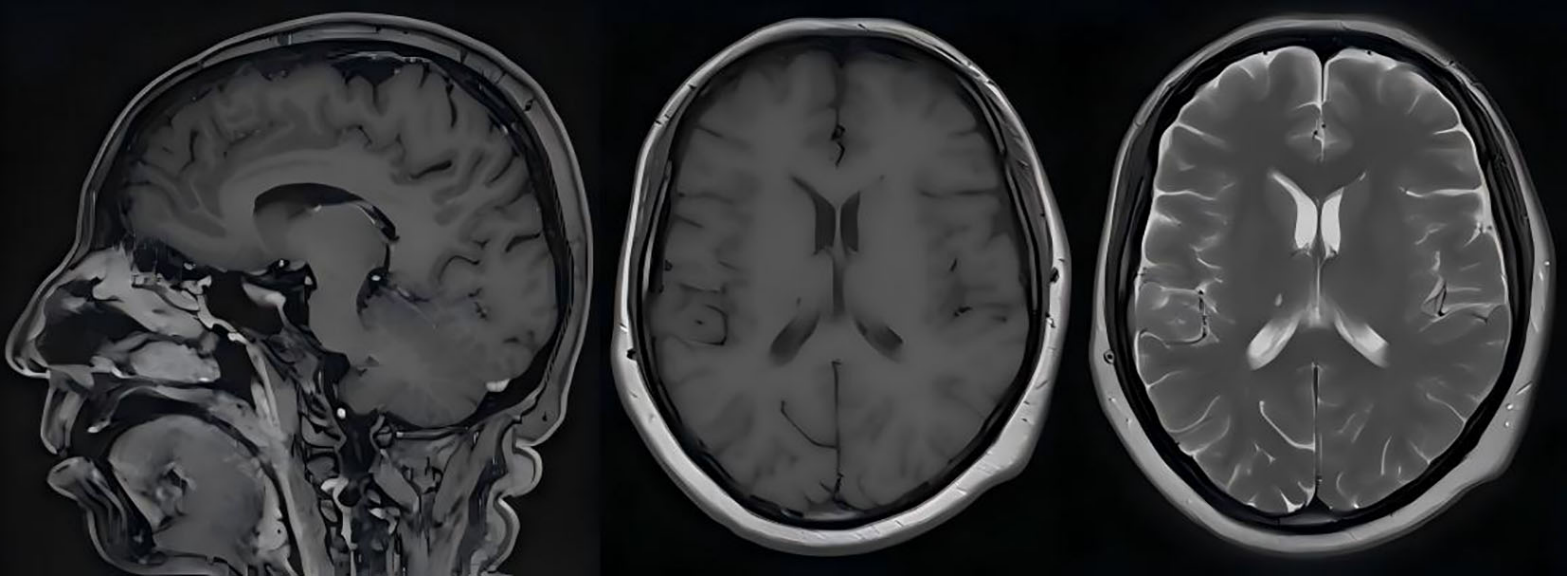
Despite T1, T2 magnetic resonance imaging with contrast of the head showed no signs of meningeal metastasis. On May 26, 2025, the patient suffered a seizure.

**Figure 4:**
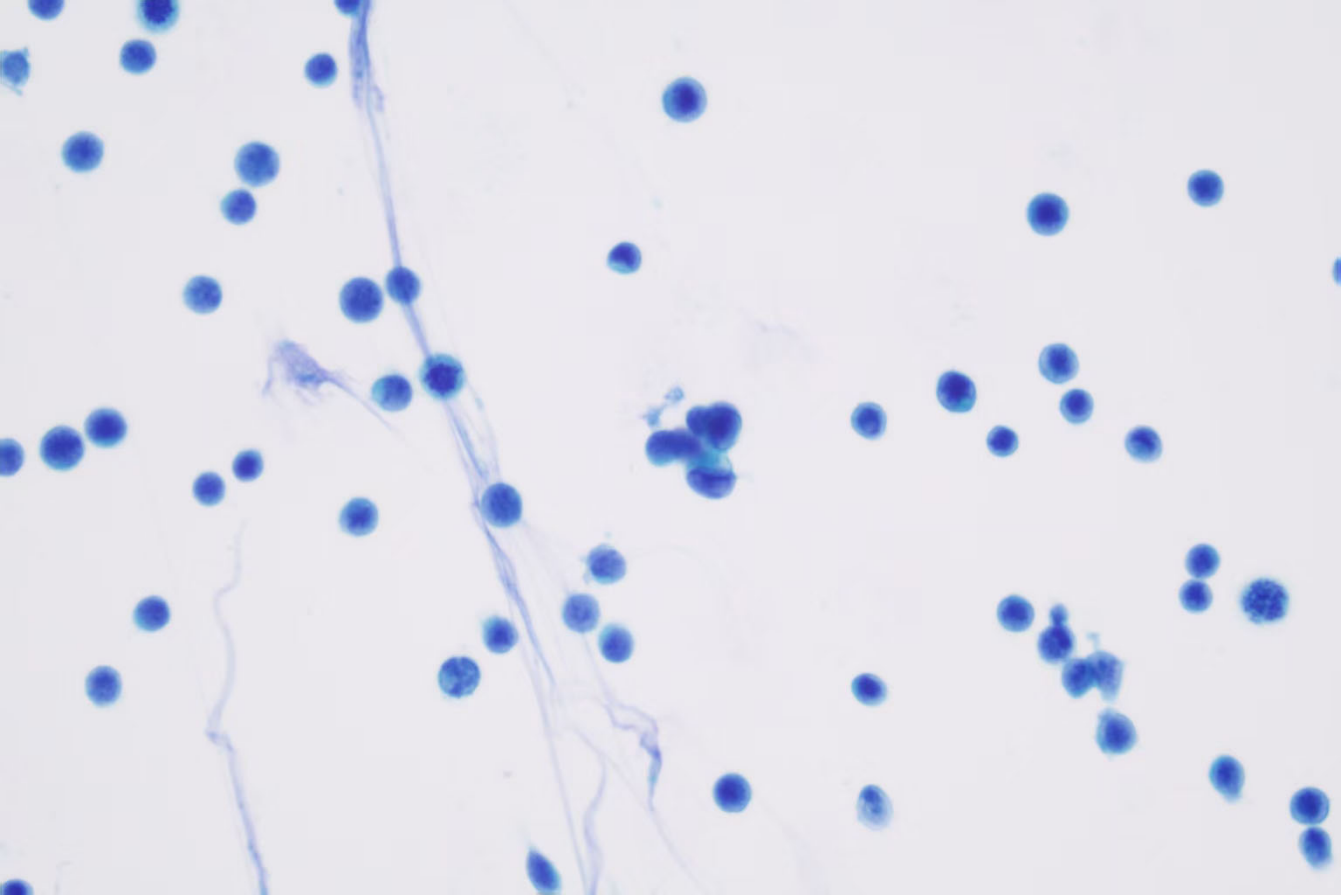
Cerebrospinal fluid (CSF) obtained via lumbar puncture (Thinprep) revealed markedly enlarged atypical cells aggregating in a small, tight cluster with finely vacuolated cytoplasm.

At 2 weeks after completion of radiotherapy, the patient experienced a transient unconsciousness, severe headache, nausea, and vomiting. She was readmitted to our hospital on May 25, 2025. Neck stiffness is present, and the meningeal irritation sign is positive. Additionally, the patient suffered a seizure due to symptom aggravation. Conventional biochemical examinations including routine blood, urine, liver, and kidney function tests were normal. MRI showed no abnormalities (**Figure 3**). The serum levels of tumor markers were 171.6 U/mL cancer antigen 199 (CA199), 11.2 U/mL cancer antigen 125 (CA125), 9.29 ng/mL neuron-specific enolase (NSE), and 2.63 ng/mL cytokeratin. Results from a lumbar puncture revealed that the patient’s CSF was colorless, the intracranial pressure was 250 mmH_2_O, the protein level was 0.75 g/L, the glucose level was 1.72 mmol/L, the chloride level was 113.9 mmol/L, and the immunoglobulin IgG level was 63.1 mg/L. Tumor cells were identified within the patient’s CSF via liquid-based technology (ThinPrep TCT2000) combined with Papanicolaou staining (**Figure 4**). Based on these results, a diagnosis of leptomeningeal metastasis was made. The patient’s Karnofsky performance status (KPS) score was determined to be 40–50 points. Simultaneously, the patient presented with hypopituitarism, and no lesions were seen on MRI. Consequently, immune-related hypophysitis was suspected, and it was advised to discontinue the administration of cadonilimab.

The patient’s treatment regimen included oral temozolomide (350 mg on days 1–5) orally every 3-week cycle. Simultaneous administration of intrathecal chemotherapy using pemetrexed disodium (PD, 50 mg) and dexamethasone (5 mg) was performed once a week. Three cycles of intrathecal chemotherapy and one cycle of oral temozolomide markedly alleviated the patient’s symptoms, and his KPS score increased to 60 points. The patient declined further treatment for personal reasons and was discharged from the hospital. After 2 months, the patient began to experience headaches and epileptic seizures and succumbed due to disease progression. The patient’s overall survival (OS) time was 24 months, and the patient had survived for 4 months from the time of diagnosis of leptomeningeal metastasis.

**Figure 5:**
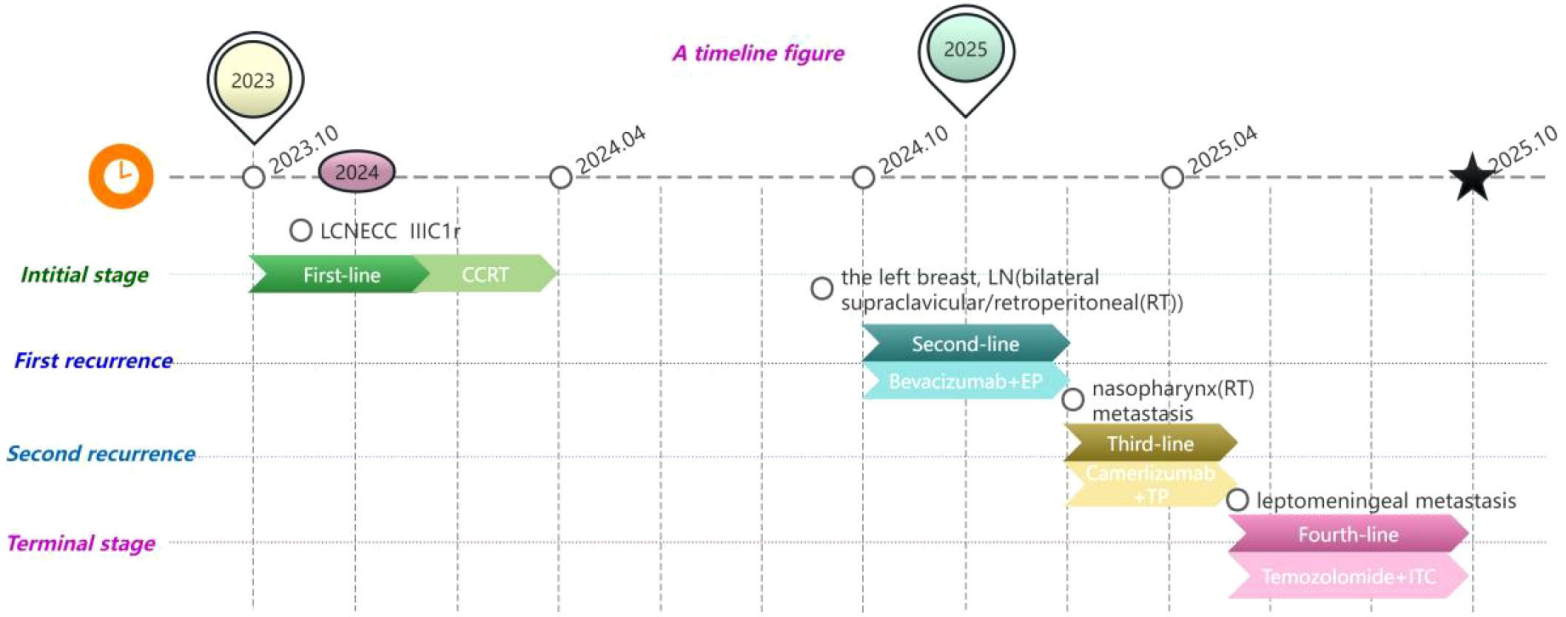
LCNECC, large cell neuroendocrine carcinoma of the cervix; CCRT, concurrent chemoradiotherapy; LN, lymph node; RT, radiotherapy; EP, etoposide + platinum; TP, paclitaxel + platinum; ITC, intrathecal chemotherapy.

The chronology of the patient’s clinical course, including all major events and treatments, is summarized in **Figure 5**.

## Discussion

NECC is a rare entity, constituting approximately 0.9%–1.5% of all cervical malignancies, and is associated with a poor prognosis (12–15). Management of patients with NECC remains complex due to the overall rarity and lack of established treatment algorithms and prospective studies in the literature (16–18). NECC frequently metastasizes to the lungs, liver, and bones (19, 20). However, LM from LCNECC is extremely rare, with only five cases of LM in NECC having been reported (7–11). The cytological features of malignant cells in cerebrospinal fluid serve as the gold standard for diagnosing LM (5, 21). Previous case reports have described the use of imaging as well as plasma and CSF EBV DNA testing to diagnose LM (22). Since negative MRI findings are not exclusionary, the second modality, CSF cytology, is an essential layer in the diagnosis of this pathology (23). The diagnostic reasoning process for this patient was as follows: The patient presented with progressively worsening headache, nausea, vomiting, and epileptic seizures, suggestive of meningeal irritation. Although no significant soft meningeal enhancement or nodular lesions were observed on cranial MRI with contrast, we decided to perform a lumbar puncture based on the following clinical considerations:

- Highly suggestive clinical presentation: The patient’s neurological symptoms progressively deteriorated, manifesting as typical meningeal irritation signs (headache, nausea, vomiting) combined with epileptic seizures, which closely align with the clinical pattern of soft meningeal metastasis. Literature reports indicate that approximately 10%–15% of LM cases may present with negative or inconclusive MRI findings at initial diagnosis (23), while cerebrospinal fluid cytology can enhance diagnostic sensitivity.
- Systematic exclusion of other etiologies: Infectious meningitis was deemed unlikely due to the lack of systemic signs of infection and repeatedly negative CSF microbiological studies. Paraneoplastic syndromes, while plausible given the primary malignancy, were less consistent with the rapid, neurological progression and the eventual cytological confirmation of malignant cells in CSF. Treatment-related neurotoxicity from prior therapies (e.g., platinum-based chemotherapy) was also evaluated. However, the clinical presentation with meningeal signs and cranial neuropathies, coupled with the CSF (elevated protein with malignant cytology) (24), argued against a primary toxic etiology. In terms of other causes of malignant meningitis, the patient had previously confirmed nasopharyngeal metastases, which are anatomically adjacent to the skull base meninges, increasing the risk of direct tumor dissemination to the subarachnoid space.
- Driver of high-risk clinical context: The patient had a history of nasopharyngeal metastases (an extremely rare metastatic site), with progressive elevation of serum tumor marker NSE, suggesting the active progression of neuroendocrine tumors. Under this clinical context, even with negative MRI findings, the pre-test probability of LM remains significantly elevated. Therefore, lumbar puncture is clinically justified when repeated cranial MRI results are negative but the patient exhibits progressively worsening meningeal irritation symptoms. This diagnostic strategy ultimately confirmed LM through the detection of malignant cells in CSF, demonstrating that cerebrospinal fluid examination can precede imaging findings in highly clinically suspected cases.

The clinical features and results of each case are presented (**Table 1**). The patients’ ages at diagnosis ranged from 33 to 54 years, with a median age of 44 years. The histological subtypes were as follows: one case of LCNECC, one case of SCNECC, and three unclassified cases. LM diagnosis typically manifests as an advanced clinical feature following disease dissemination. However, it may occur at any clinical stage. Two cases developed at the IB2 stage and one at the IVB stage. One IVB-stage patient presented with LM at the initial sign. In initial treatment, two underwent surgery for cervical cancer, while two received concurrent chemoradiotherapy (CCRT) (**Table 1**). Four cases presented with metastases to the lungs, liver, pancreas, bones, breasts, mediastinum, pelvic, and retroperitoneal lymph nodes as well as the brain parenchyma prior to the diagnosis of LM. Prior to LM, our patient presented with metastases to the left breast, supraclavicular lymph nodes, and retroperitoneal lymph nodes. Pathological examination verified the rare metastasis in the nasopharynx. While the median time to the occurrence of LM is typically 8 months post-diagnosis, ranging from 3 to 19 months, our patient experienced this complication notably later, at 20 months post-diagnosis.

**Table 1:** Similar cases of NECC with LM.

Case	Year	Age	Stage	Pathology	Initial treatment	Recurrences prior to LM	Signs	Diagnosis	Past history of parenchymal brain metastasis	Time to Lm diagnosis	Treatment for Lm	Survival after LM diagnosis	Overall survival (OS)
Kumar (9)	2004	39	IVB	LCNECC	N/A	None	Headache, facial palsy, hearing loss, dysphagia, dysarthria	Gd-MRI	No	Initial sign	N/A	N/A	N/A
Asensio (7)	2009	54	IB2	NECC	CCRT, surgery	Liver, brain, bone, mediastinum	Lumbar pain, paraparesis	MRI	Yes	14 months	RT (WB, L1–L3, C6–C7 spine)	7 months	21 months
Komiyama (8)	2011	33	IB2	NECC	CT, surgery	Breast, lung, pancreatic, LN (mediastinal)	disorientation, convulsions, and impaired abduction of the right eye	CT/Autopsy	No	19 months	RT (WB)	10 days	19 months
Watanabe (11)	2012	39	IVB	SCNECC	sCT (paclitaxel carboplatin)	LN (pelvic, paraaortic), sacrum	Headache, nausea, vomiting	MRI	Yes	3 months	No	4 week	4 months
Patterson (10)	2023	42	N/A	NECC	CCRT (cisplatin/etoposide)	LN (pelvic, retroperitoneal) brain	Headache	CSF/MRI	Yes	13 months	ITC (topotecan)+RT (WB, cervical, and lumbar spine)	2 months	15 months
Our patient	2023	49	IIIC1r	LCNECC	CCRT (cisplatin/etoposide)	Breast, nasopharynx, LN (supraclavicular, retroperitoneal	Headache, nausea, vomiting	CSF	No	20 months	ITC (pemetrexed disodium) + temozolomide	4 months	24 months

The possible mechanisms of LM have been considered in the following four ways: (1) Hematogenous metastasis, which may be the most common route of tumor metastasis (25), (2) direct extension by pre-existing brain metastases, (3) direct invasion by subdural or epidural tumors, and (4) direct spread from adjacent areas outside the CNS (4, 5, 26). In **Table 1**, three cases (case 1, case 2, and case 5) may be direct extensions of LM due to brain or spinal cord metastases. One case (case 4) may have been invaded by sacral metastases, and another case (case 3) may have been extended by hematogenous dissemination following lung metastasis. Our patient’s LM may be invaded by the adjacent area of the nasopharynx. This is based on its unique metastatic pattern: nasopharyngeal metastases appear before LM, and no other brain parenchymal or spinal cord lesions were observed on imaging. Currently, there are reports of secondary LM after radiotherapy and chemotherapy for nasopharyngeal carcinoma (27). Certainly, this is the first report of cervical cancer with nasopharyngeal metastases prior to LM.

Patients may manifest a diverse array of clinical symptoms as a result of LM involving the cerebral hemispheres, cranial nerves, or spinal cord and nerve roots (3, 28). The most common clinical manifestations of meningeal metastasis in the cervical region include headache (three cases, 60%), impaired right-eye abduction function (one case, 20%), auditory neurological symptoms such as hearing loss and tinnitus (one case, 20%), and spinal-nerve-related symptoms like low back pain and sensory disturbances. Sensory loss due to sciatic nerve injury was also experienced (one case, 20%). Our patient presented with symptoms such as headache, nausea, vomiting, and epileptic seizures.

LM is a life-threatening complication associated with malignant tumors, and it has a poorer prognosis compared to brain metastases. Due to the paucity of clinical trial data, the treatment for LM is mainly guided by expert recommendations. Whole-brain radiotherapy (WBRT), delivered at a dose of 30–40 Gy over two to three fractions, is recommended as it can reduce tumor volume, promote cerebrospinal fluid circulation, and alleviate hydrocephalus (29, 30). Intrathecal chemotherapy is also a viable option because there are no blood–brain barrier limitations (31). A phase II clinical trial demonstrated the efficacy and safety of pembrolizumab in patients with solid tumor lymphoma, with a central nervous system response rate of 38% and acceptable safety (32). Another phase II trial involving 18 patients with LM receiving combined ipilimumab and nivolumab treatment reported a 44% overall survival rate at 3 months (33). Unfortunately, our patient with PD_L1 (CPS = 5), who was treated with cadonilimab subsequently, had to discontinue the drug due to immune-related pituitary inflammation.

Given the patient’s refusal of whole-brain radiotherapy and the impracticality of immunotherapy due to toxicity, our patient received intrathecal pemetrexed disodium treatment along with oral temozolomide. Pemetrexed is a multi-target anti-folate metabolism drug; intrathecal pemetrexed has emerged in recent years as a potential option, primarily based on small retrospective series and case reports demonstrating its feasibility and some evidence of clinical or cytological response in LM from non-small cell lung cancer (NSCLC) and other solid tumors (34, 35). The LM data of LCNECC are completely lacking; the systemic activity of pemetrexed in neuroendocrine tumors provides indirect theoretical support for its application. The addition of concurrent oral temozolomide (24, 36, 37) was based on a dual mechanism of action aimed at both the meningeal compartment and potential parenchymal disease. Temozolomide, an alkylating agent with good central nervous system penetration, was intended to provide low-grade, continuous systemic anti-tumor activity that might synergize with the focal, high-concentration effect of intrathecal pemetrexed. The selection of intrathecal pemetrexed combined with oral temozolomide for this patient was guided by a palliative intent and the dire need for symptom control in the face of a universally poor prognosis. This approach must be explicitly framed as exploratory, given the absence of prospective, randomized evidence for any regimen in LM from LCNECC.

Current research indicates that neither radiotherapy nor chemotherapy alone can improve overall survival (OS) in patients with LM (38–42). In **Table 1**, 60% (3/5) of the patients received localized radiotherapy, whereas 40% (2/5) of the patients had no treatment. Only 20% (1/5) were given intrathecal chemotherapy in combination with radiotherapy (WB, cervical, and lumbar spine), but this method did not lead to significant improvements in OS. The patient in this case refused radiotherapy and was treated with intrathecal pemetrexed combined with oral temozolomide. Although the treatment was technically feasible and tolerable, the overall prognosis remained extremely poor, and the patient died 4 months after the diagnosis of LM. Regarding treatment, this case only provides the following limited conclusions: Intrathecal pemetrexed combined with oral temozolomide is technically feasible, and no severe acute toxicity was observed during administration. The treatment may have achieved short-term symptom control (the patient’s headache and nausea symptoms were temporarily alleviated), but objective efficacy could not be assessed due to the lack of imaging follow-up and survival data. The effectiveness of this regimen has not been fully confirmed, and it should not be recommended as standard therapy given the patient’s short-term mortality.

We report the rare case of a patient with LCNECC with LM, in which nasopharyngeal metastasis occurred prior to LM. The computed CT and MRI scans failed to yield evidence of leptomeningeal, brain parenchymal, or spinal cord metastases. However, CSF was conducted to diagnose LM.

## Patient’s perspective

During the patient’s treatment process, she demonstrated remarkable resilience. Initially, she tolerated chemoradiotherapy with manageable fatigue and harbored hopes for a cure. After the first recurrence, she was anxious yet remained committed to second-line therapy. As the disease disseminated to the nasopharynx and leptomeninges, her physical burden escalated, presenting symptoms such as dysphagia, headache, and neurological deficits. The multidisciplinary team, the patient, and her family engaged in in-depth discussions for each new therapeutic approach, and her objective was to prolong her life while maintaining quality. After the diagnosis of leptomeningeal metastasis, in accordance with her and her family’s wishes, the focus of care shifted to comfort and symptom control through intrathecal chemotherapy and supportive measures rather than less effective systemic anti-tumor therapies. The patient’s family gave their consent to share this case to illustrate the challenges encountered by patients with aggressive carcinomas and the significance of empathetic communication and shared decision-making in the treatment of such complex diseases.

## Data Availability

The original contributions presented in the study are included in the article/supplementary material. Further inquiries can be directed to the corresponding author.
